# Physiological Adaptation of Three Wild Halophytic *Suaeda* Species: Salt Tolerance Strategies and Metal Accumulation Capacity

**DOI:** 10.3390/plants11040537

**Published:** 2022-02-17

**Authors:** Farag Ibraheem, Ateeq Al-Zahrani, Ahmed Mosa

**Affiliations:** 1 Biology and Chemistry Department, Al Qunfodah University College, Umm Al-Qura University, Al Qunfodah 21912, Saudi Arabia; aaalzahrani@uqu.edu.sa; 2 Botany Department, Faculty of Science, Mansoura University, Mansoura 35516, Egypt; 3 Soils Department, Faculty of Agriculture, Mansoura University, Mansoura 35516, Egypt

**Keywords:** *Suaeda*, salinity, physiology, oxidative stress, potential toxic elements, betacyanin, carbon, nitrogen, phytoremediation

## Abstract

Understanding salt tolerance mechanisms in halophytes is critical for improving the world’s agriculture under climate change scenarios. Herein, the physiological and metabolic responses of *Suaeda monoica*, *Suaeda vermiculata*, and *Suaeda schimperi* against abiotic stress in their natural saline environment on the east coast of the Red Sea were investigated. The tested species are exposed to different levels of salinity along with elemental disorders, including deficiency in essential nutrients (N&P in particular) and/or elevated levels of potentially toxic elements. The tested species employed common and species-specific tolerance mechanisms that are driven by the level of salinity and the genetic constitution of *Suaeda* species. These mechanisms include: (i) utilization of inorganic elements as cheap osmotica (Na+ in particular), (ii) lowering C/N ratio (*S. monoica* and *S. schimperi*) that benefits growth priority, (iii) efficient utilization of low soil N (*S. vermiculata*) that ensures survival priority, (v) biosynthesis of betacyanin (*S. schimperi* and *S. vermiculata*) and (vi) downregulation of overall metabolism (*S. vermiculata*) to avoid oxidative stress. Based on their cellular metal accumulation, *S. monoica* is an efficient phytoextractor of Cr, Co, Cu, Ni, and Zn, whereas *S. vermiculata* is a hyper-accumulator of Hg and Pb. *S. schimperi* is an effective phytoextractor of Fe, Hg, and Cr. These results highlight the significance of *Suaeda* species as a promising model halophyte and as phytoremediators of their hostile environments.

## 1. Introduction

Coastal salt marshes are transition zones between land and sea and act as natural buffers against deteriorative impacts of saltwater intrusion, coastal erosion, and contaminants release [1]. These regions usually contain large levels of salinity along with substantial amounts of potentially toxic elements (PTEs) as a result of various anthropogenic activities (e.g., rapid urbanization, marine construction, oil spilling, domestic waste dumping, landfilling due to the advancement of a seaside framework, brine discharge from desalination plants and agricultural practices [2]. Climate change is expected to increase temperature and evapotranspiration and thus can aggravate salinity and PTE-induced stress, particularly in arid and semi-arid regions [3]. Such harsh conditions of salinity and PTEs in arid salt marshes restrict plant vegetation to halophytic plants, which evolved exceptional ability to grow and reproduce in a highly saline environment [1,4]. Interestingly, the ongoing increase in atmospheric CO_2_ can improve the salinity tolerance of C3 and C4 halophytes [5]. Therefore, these unique plants can contribute significantly to carbon sequestration and thus can reduce the impact of global climate change [1]. Halophytes can further be used as intercropping and rotating species to improve crops’ productivity, given their high potential to desalinize the high salt accumulations [6]. These features highlight the potentiality of halophytes as promising biological resources for improving the world’s agriculture in the climate change scenario via genetic and biotechnological approaches. Intensive research has been undertaken for a better understanding of the salt tolerance mechanisms in glycophytes and halophytes. However, the full picture, particularly in halophytes, is far from clear.

To cope with salinity-induced challenges, halophytes employ common as well as species-specific mechanisms to minimize their detrimental effects. Common salt tolerance mechanisms include (i) regulation/compartmentalization of ions (Na^+^ and Cl^−^) uptake and localization in vacuoles [7], (ii) accumulation of organic osmolytes in the cytoplasm to balance the osmotic effects in vacuoles [8,9], and (iii) maintaining a balance between the production of reactive oxygen species (ROS) and the total quenching activity of antioxidative system [10]. Species-specific mechanisms may include succulence, extrusion of toxic ions, special anatomical structures (hairs, salt glands), redistribution of excessive ions to senescent leaves [11,12], and synthesis of stress-related pigments with specific physiological functions such as betacyanin [13]. Activation of the above tolerance mechanisms involves the diversion of a significant portion of essential plant metabolites such as carbohydrates and nitrogenous compounds away from biomass production [14]. Such metabolic shunting drives a trade-off between halophyte growth and survival: responses that differ among taxa and are not fully understood [15]. Along with their excess salt ions, halophytic habitats are enriched with PTEs. To cope with the adverse effects of such PTEs, halophytes employ various mechanisms, including metals stabilization in the root zone, complexation with root exudates, changing the metal ions, precipitation as insoluble deposits inside vacuoles, and establishing a partnership with heavy metal tolerant soil microorganisms [16,17].

Halophytes belong to different angiosperms plant families, suggesting a polyphyletic origin of salt tolerance [18]. Among these families, Amaranthaceae (previously known as Chenopodiaceae) is an interesting example as it contains the largest number of known halophytes with high capabilities of salt tolerance [18]. *Suaeda* is one of the extreme obligate halophytic chenopods and has been proposed as a model system for the dissection of salt tolerance in halophytes [18]. *Suaeda* species are generally perennials chamaephyte (dwarf-shrub) with succulent leaves. They are distributed in various saline habitats with different salinity levels and exhibit differential capabilities of withstanding high salinity levels ranging from 200 mM to 400 mM or even more [19]. Along with their significance as model plants for dissection of salt tolerance, they have been suggested as promising biological tools for desalinization of hypersaline lands because of their high capacity of salt uptake and accumulation [20]. In fact, some *Suaeda* species can remove more than two tons of salt/hectare in a single harvest [21,22]. In addition, *Suaeda* species have been acknowledged for their high efficiency as phytoremediators with the ability to uptake substantial amounts of PTEs [23]. These halophytic species are adapted to overcome PTEs accumulation similar to glycophytes [24]. The salt stress tolerance mechanisms of *Suaeda* species, similar to most other dicotyledonous halophytes, mainly depend on the accumulation of Na^+^ and Cl^−^ in leaves [25,26], where their succulence enables the dilution of ions concentration and thus alleviate ions toxicity [27]. Furthermore, osmolytes such as glycine betaine, proline, and sugars play key roles in osmotic adjustment in some *Suaeda* species [28,29]. Further, non-enzymatic antioxidants such as flavonoids and phenolics, along with antioxidant enzymes, contribute to their adaptation against salinity-induced oxidative stress [10,30].

The east coast of the Red Sea is a typical hyper-arid saline region with high temperature, limited precipitation, high salinity, and vulnerability to contamination derived from oil trading and other anthropogenic activities developing along the Red Sea coast. It is particularly rich in *Suaeda* species. Examples include *S. egyptiaca*, *S. fruticosa*, *S. monoica*, *S. vermiculata*, *S. pruinosa*, and *S. schimperi* [31]. The current harsh climatic conditions in the region, as well as the predicted climate change-induced increase in temperature and evapotranspiration, are expected to exacerbate salinity-induced deleterious effects on *Suaeda* growth and physiology in the region [1,32]. Up to our knowledge, the differences in the activity and the relative contribution of the above individual mechanisms to salt tolerance in *Suaeda* species in the area have not been reported. In addition, the species/habitat variations in *Suaeda* species seem to depend on differential efficiency of salt tolerance mechanisms among these species, which may be associated with different adaptive physiological and molecular mechanisms in response to different levels of soil salinity [33].

In the current study, three species of the genus *Suaeda* including *Suaeda monoica* Forssk. ex J.F.Gmel., *Suaeda vermiculata* Forssk. ex J.F.Gmel., and *Suaeda schimperi* Moq. that dominate three separate salt marshes at different vicinity to the east coast of the Red Sea were selected. These species are genetically related but differ in their leaf reddening phenotype, which has been reported as an adaptive strategy for salt tolerance [34]. The aim of the current study was to assess the impact of the interaction between different levels of soil salinity and the differential physiological responses of the selected *Suaeda* species, if any, on their successful adaptation in particular salt marshes. Herein, the hypothesis is that the successful adaptation of different *Suaeda* species in the selected salt marshes is shaped by the interplay between the magnitude of salinity in their rhizospheric soil and their relative salt tolerance evolutionary strategies. In addition, common and species-specific physiological responses may operate among species. Therefore, performing a comparative analysis of the physiological responses of these genetically related species against the physicochemical properties of their rhizospheric soil would be a useful approach to gain insights on possible common and species-specific tolerance mechanisms in these species.

The specific objectives of this study are to (i) monitor the levels of soil salinity, nutrients status, and PTEs concentration in the rhizospheric soil of the tested *Suaeda* species, (ii) determine water and nutrient status of *Suaeda* species as affected by physicochemical properties of soil, (iii) explore critical biochemical indicators of *Suaeda* species relevant to their physiological adaptation, and (v) evaluate the bioaccumulation capacity of PTEs by *Sauuda* species for the future phytoremediation planning.

## 2. Results

### 2.1. Soil Physicochemical Properties

Sand was the dominant component among soil fractions with a higher silt and clay content in soil supporting *S. vermiculata* (Table 1). The texture was sandy in soils supporting *S. monoica* and *S. schimperi*; however, it was sandy clay loam in the soil supporting *S. vermiculata*. The soil supporting *S. vermiculata* had higher porosity (49.93%) than both *S. monoica* (39.98%) and *S. schimperi* (43.89%). Water holding capacity in all soils was generally low (31.98–38.15%), with a relative superiority of soil supporting *S. vermiculata* (Table 1).

The tested soils were all alkaline, with pH values between 7.78 and 8.65 (Table 1). The EC values in soils supporting *S. vermiculata* (18.37 dSm^−1^) and *S. schimperi* (16.25 dSm^−1^) were significantly higher than soil supporting *S. monoica* (5.04 dSm^−1^) (Table 1). Furthermore, soil supporting *S. vermiculata* and *S. schimperi* had comparable levels of calcium carbonate (0.79%), which were higher than soil supporting *S. monoica* (0.55%). The significantly high EC values of soils supporting *S. vermiculata* and *S. schimperi* were associated with higher water-soluble Cl^−^, Na^+^, and K^+^ ions. In addition, the high pH value of soil supporting *S. vermiculata* was correlated with high soluble HCO_3_^−^ concentration (6.35 Cmol/100 g). The soil supporting *S. schimperi* had about two- and six-fold greater Mg^2+^ concentration (10.42 Cmol/100 g) than soils supporting *S. vermiculata* and *S. monoica*, respectively (Table 1).

Regarding the nutrient status of the tested soils, available phosphorus in soil supporting *S. vermiculata* (9.40 mg kg^−1^) was higher than those supporting *S. schimperi* (6.53 mg kg^−1^) and *S. monoica* (8.17 mg kg^−^^1^) (Table 2). The average concentrations of K^+^ in rhizospheric soils of *S. schimperi*, *S. vermiculata*, and *S. monoica* were 383.8, 427.8, 178.8 mg kg^−1^; whereas the corresponding values were 1519.1, 1229.9 and 1441.0 mg kg^−1^ for Ca^2+^ and 1250.2, 679.4, and 215.3 mg kg^−1^ for Mg^2+^. Soil organic elements were generally low. Carbon was not detected in soil supporting *S. monoica*, recorded low value in soil supporting *S. vermiculata* (0.23%), and was relatively high in soil supporting *S. schimperi* (1.95%). Nitrogen and sulfur were not detected in soils supporting *S. vermiculata* and *S. monoica* and showed low values in soil supporting *S. schimperi* (0.18 and 0.47%, respectively).

### 2.2. PTEs Concentration in the Tested Soils and Their Ecological Risk Assessment

Soil available contents of PTEs varied among locations and were ranked based on their average values (mg kg^−1^) as Mn (18.21), Fe (7.94), Pb (4.71), Zn (3.62), Cu (2.26), Hg (1.55), Ni (0.71), Cr (0.333), Co (0.326), and Cd (0.07) (Table 3). Soil supporting *S. schimperi* had the highest levels of Cd, Co, Cu, Fe, and Mn. Meanwhile, the highest values of Cr, Hg, and Ni were recorded in soil supporting *S. monoica*. The soil supporting *S. vermiculata*, however, exhibited the highest Pb and Zn values. These results were greatly higher than other reported values in *Typic Torripsamment* such as [35] (Cd, Cu, Fe, Mn, Ni, Pb, and Zn), [36] (Cd, Cr, Co, Mn, and Ni), and [37] (Cd, Cu, Fe, Mn, Ni, Pb, and Zn) (Table 3).

**Figure 1:**
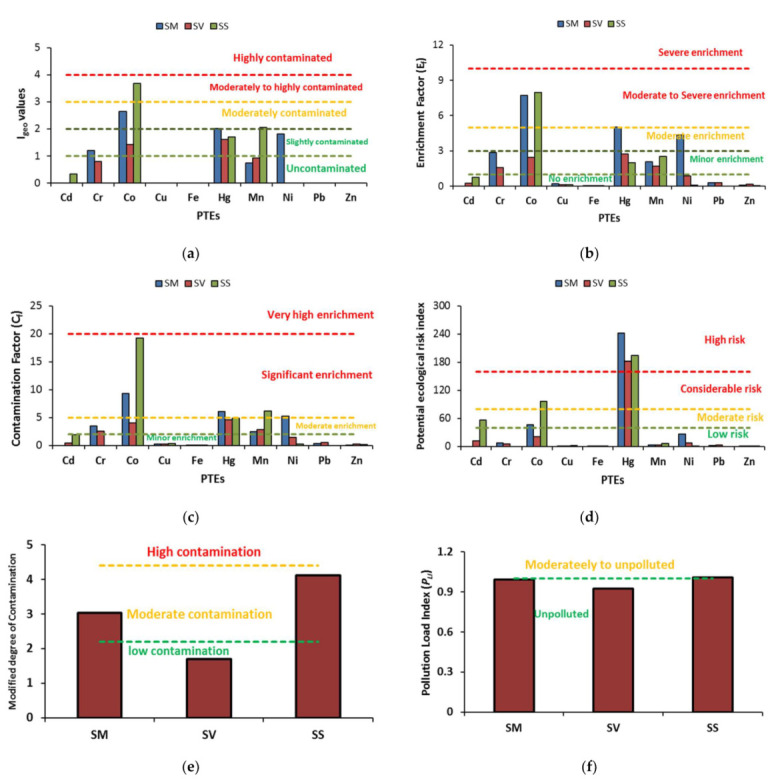
Ecological risk assessment indices of TEs in different soils supporting the tested *Suaeda* species: (**a**) geo-accumulation index; (**b**) enrichment factor; (**c**) contamination factor; (**d**) potential ecological risk index; (**e**) modified degree of contamination; and (**f**) pollution Load Index. SM: *S. monoica*, SV: *S. vermiculata*, and SS: *S. schimperi*.

Ecological risk assessments of PTEs in different locations are shown in Figure 1 and are categorized into six classes to interpret the obtained contamination levels of PTEs (Table S1, Supplementary Data). Geo-accumulation index indicated an uncontaminated effect of Cd, Cu, Fe, Pb, and Zn in all locations (0 < *I_geo_* < 1). Other toxic elements (Cr, Hg, Mn, and Ni) showed slight contamination (1 < *I_geo_* < 2). Meanwhile, Co exhibited moderate-to-high contamination (3 < *I_geo_* < 4) effect in the studied locations with a higher risk in soil supporting *S. schimperi*.

According to enrichment factor (*E_f_*), some PTEs (Cd, Cu, Fe, Pb, and Zn) exhibited no enrichment in all locations (0 < *E_f_* < 1). Other elements (Cr and Mn) recorded minor enrichment (1 < *E_f_* < 3). Mercury (Hg) and Ni showed a moderate enrichment (3 < *E_f_* < 5) in soil supporting *S. monoica*. However, cobalt showed moderate to severe enrichment (5 < *E_f_* < 10) in *S. monoica* and *S. schimperi* supporting soils.

The contamination factor (*C_f_*) index showed minor contamination (*C_f_* < 2) of Cd, Cu, Fe, Pb, and Zn. Moderate contamination (2 ≤ *C_F_* < 5) was observed with Cr (*S. monoica* and *S. vermiculata* supporting soils), Co (*S. vermiculata* supporting soil), Hg (*S. vermiculata* and *S. schimperi* supporting soils), and Mn (*S. monoica* and *S. vermiculata* supporting soils). Some PTEs reached significant contamination (5 ≤ *C_F_* < 20) including Co (*S. monoica* and *S. schimperi* supporting soils), Hg (*S. monoica* supporting soil), Mn (*S. schimperi* supporting soil), and Ni (*S. monoica* supporting soil).

Ecological risk index ($E\begin{array}{c} i\\ r \end{array}$) pointed to a low risk of most PTEs (Cr, Cu, Fe, Mn, Ni, Pb, and Zn) in all locations ($E\begin{array}{c} i\\ r \end{array}$< 40). In contrast, Hg showed a high risk (160 ≤$\;E\begin{array}{c} i\\ r \end{array}$ < 320) in all locations given its high toxicity coefficient (40). Meanwhile, Cd and Co exhibited moderate to considerable risk with higher values in *S. schimperi* supporting soils. The combined effect of PTEs hazard was explored using a modified degree of contamination and pollution load index. The modified degree of contamination showed low contamination (1.5 ≤ mCD < 2.2) with *S. vermiculata* supporting soil. However, *S. monoica* and *S. schimperi* supporting soils recorded moderate contamination (2.2 ≤ mCD < 4.4). Conversely, the pollution load index revealed an unpolluted effect (0 < *P_LI_* ≤ 1) of soils in the studied locations (Figure 1).

### 2.3. PTEs Concentration in the Tested Suaeda Species

To gain insights into the PTEs transport to the aerial parts of *Suaeda* plants, the cellular concentrations of PTEs in leaves of the tested *Suaeda* species were analyzed and compared (Table 3). Values of PTEs (mg kg^−1^) averaged as: Cd (0.50), Cr (81.98), Co (5.09), Cu (37.22), Fe (1409.15), Hg (23.05), Mn (138.22), Ni (9.11), Pb (14.28), and Zn (93.91). *Suaeda. monoica* showed the highest concentrations of Cr, Co, Cu, Ni, and Zn. *Suaeda* *schimperi*; however, had the highest values of Cd, Fe, and Mn. Meanwhile, *S. vermiculata* exhibited the highest values of Hg and Pb.

**Figure 2:**
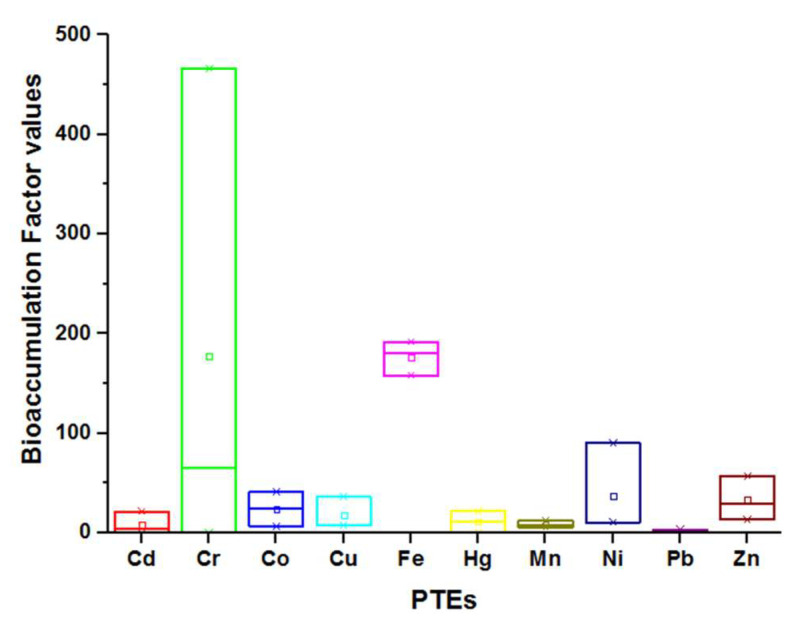
Bioaccumulation index of the tested *Suaeda* species. Values were calculated by dividing the metal concentration in *Suaeda* species by its corresponding concentration in rhizospheric soil. Plants with values higher than 1.0 are considered phytoextractors.

Our calculations of the bioaccumulation factors (BCR) of PTEs of the tested *Suaeda* species revealed high BCR values that ranged between 3.3 and 466.1 (Figure 2). These findings illustrated the potential utilization of these plants as phytoextractors since values greater than 1.0 pointed to hyperaccumulating plants; however, values below 1.0 are indicative of excluder plants. Chromium (Cr) showed the highest bioaccumulation among PTEs, but Pb showed the lowest value.

### 2.4. Levels of Inorganic and Organic Nutrients in Leaves of the Investigated Suaeda Species

Leaves of the tested *Suaeda* species varied significantly in their content of inorganic minerals (Table 4). Both *S. monoica* (1.485 mg g^−1^) and *S. schimperi* (1.275 mg g^−1^) contained significantly higher P than *S. vermiculata* (0.452 mg g^−1^). Meanwhile, Ca^2+^ concentrations in *Suaeda* species were comparable (10.38–12.63 mg g^−1^). Likewise, Mg^2+^ concentrations were relatively similar in *S. schimperi* and *S. vermiculata* (6.63 mg g^−1^), and both were significantly higher than *S. monoica* (4.99 mg g^−1^). The concentrations of K^+^ were 10.04, 12.69, 10.55 mg g^−1^ DWT in leaves of *S. monoica, S. vermiculata*, and *S. schimperi*, respectively, whereas their corresponding concentrations of Na^+^ were 11.01, 17.84, and 16.19 mg g^−1^ DWT. Given the accumulation patterns of both Na^+^ and K^+^ in the tested species, *S. schimperi* had the highest Na^+^/K^+^ ratio (1.536), whereas *S. monoica* had the lowest ratio (1.097). *S. vermiculata* had intermediate Na^+^/K^+^ ratio (1.407). *Suaeda monoica* had greater leaf C content (332.6 mg g^−1^) than *S. schimperi* (265.16 mg g^−1^) and *S. vermiculata* (245.76 mg g^−1^). Similarly, *S. monoica* and *S. schimperi* had total leaf N of 32.04 mg g^−1^ and 29.79 mg g^−1^, respectively, and both were significantly higher than *S. vermiculata* (11.96 mg g^−1^) (Table 4). Given the observed differences in their total leaf C and N, the tested *Suaeda* species differed significantly in their C/N ratio. *Suaeda vermiculata* had the highest C/N ratio (20.99), whereas both *S. monoica* and *S. schimperi* had C/N values of 10.24 and 9.09, respectively. Leaves of *S. monoica* and *S. vermiculata* had relatively similar levels of Sulfur (S), whereas its level in *S. schimperi* was below the detection limit.

### 2.5. Variation in Photosynthetic Pigments and Carbohydrate Synthesis

**Figure 3:**
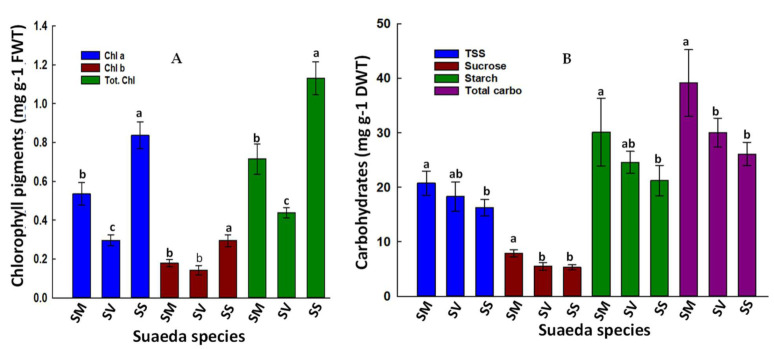
Leaf photosynthetic pigments and C assimilation: (**A**) Photosynthetic pigments: Chlorophyll a (Chl a), Chlorophyll b (Chl b), Total Chlorophyll (Tot. Chl); (**B**) Carbohydrates: Total soluble sugars (TSS), sucrose, starch, total carbohydrates. Shown are the mean values of three biological replicates. Means with the same letter are not significantly different at the probability level of 5% according to LSD. SM: *S. monoica*, SV: *S. vermiculata*, and SS: *S. schimperi*.

Because of their importance as functional and responsive traits to salinity conditions, photosynthetic pigments were measured and compared among species (Figure 3). *Suaeda schimperi* leaves contained the highest concentration of Chl a (0.84 mg g^−1^ FWT), whereas *S. vermiculata* had the lowest (0.30 mg g^−1^ FWT) among species. *Suaeda monoica* had intermediate Chl a concentration (0.54 mg g^−1^ FWT). Relatively comparable statistical relations were obtained for Chl b and total Chl (Figure 3A). Our analysis of carbohydrate residues revealed consistently higher mean values of total soluble sugars (TSS), sucrose, starch, and total carbohydrates in *S. monoica* than the other two species. *Suaeda schimperi* maintained the lowest mean values of carbohydrate residues among species (Figure 3B).

### 2.6. Changes in Total Soluble Proteins and Amino Acid Profiles

**Figure 4:**
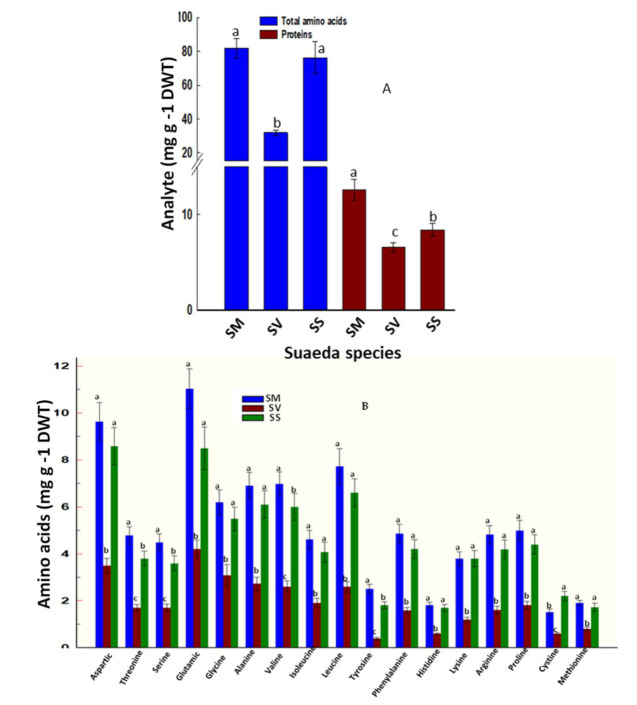
Leaf amino acids and total soluble proteins; (**A**) Total amino acids and total soluble proteins; (**B**) Amino acid profiles. Shown are the mean values of three biological replicates. Means with the same letter are not significantly different at the probability level of 5% according to LSD. SM: *S. monoica*, SV: *S. vermiculata*, and SS: *S. schimperi*.

The tested species varied significantly in their soluble proteins and amino acids content. *Suaeda monoica* had greater leaf soluble protein (12.61 mg g^−1^ DWT) than *S. schimperi* (8.30 mg g^−1^ DWT) and *S. vermiculata* (6.65 mg g^−1^ DWT) (Figure 4A). Clear differences in leaf total amino acids were noted among species. *Suaeda monoica* (83.10 mg g^−1^ DWT) and *S. schimperi* (73.32 mg g^−1^ DWT) had more than two-fold greater total amino acids than *S. vermiculata* (30.29 mg g^−1^ DWT) (Figure 4A). No qualitative differences were observed in amino acid profiles among species, yet significant differences in the relative contribution of each amino acid to the amino acids pool of the tested species were noted (Figure 4B). *Suaeda vermiculata* consistently had the lowest concentrations of individual amino acids among species. Compared to its individual amino acid concentrations, the fold change of the corresponding amino acids ranged from 2.2 to 6.0 in *S. monoica* and from 2.05 to 4.0 in *S. schimperi*. Glutamic and aspartic acids dominated the amino acid pool across species. *Suaeda monoica* and *S. schimperi* had a relatively similar ranking pattern of amino acids within the pool; however, such a pattern was significantly disturbed in *S. vermiculata*. Interestingly, *S monoica* accumulated significantly higher proline (4.71 mg g^−1^ DWT) than *S. schimperi* (4.15 mg g^−1^ DWT) and *S. vermiculata* (1.68 mg g^−1^ DWT). These findings were coordinated with similar results of phenylalanine in *S. monoica* (4.62 mg g^−1^ DWT), *S. schimperi* (3.95 mg g^−1^ DWT), and *S. vermiculata* (1.49 mg g^−1^ DWT) (Figure 4B).

### 2.7. Alterations in Oxidative Stress and Antioxidants Secondary Metabolites

**Figure 5:**
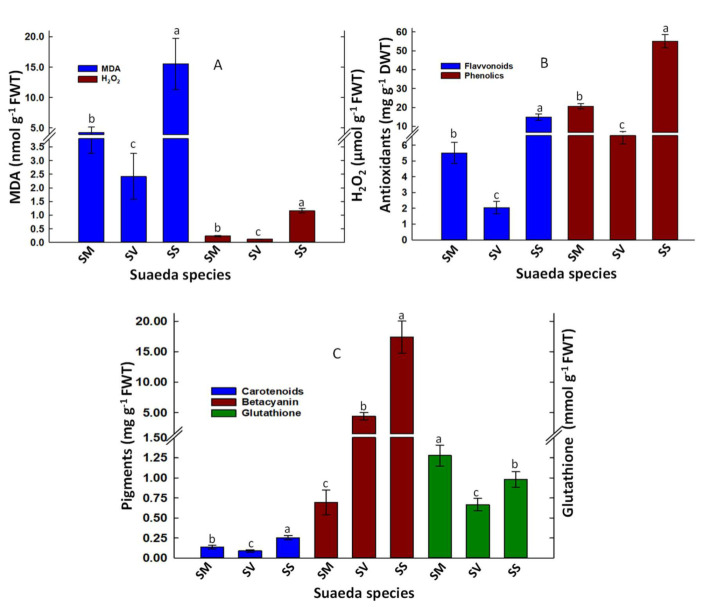
Oxidative stress and antioxidants indicators: (**A**) lipid peroxidation (Malondialdehyde; MDA) and hydrogen peroxide (H_2_O_2_); (**B**) Total flavonoids, total phenolic, and reduced glutathione; (**C**) antioxidant pigments (carotenoids and betacyanin). Shown are the mean values of three biological replicates. Means with the same letter are not significantly different at the probability level of 5% according to LSD. SM: *S. monoica*, SV: *S. vermiculata*, and SS: *S. schimperi*.

Lipid peroxidation (Malondialdehyde, MDA) and H_2_O_2_ were monitored to test salinity- and PTEs-induced oxidative stress in *Suaeda* species (Figure 5). Measurements of leaf MDA revealed unexpectedly greater MDA concentration (15.55 nmol g^−1^ FWT) in *S. schimperi* than *S. monoica* (4.20 nmol g^−1^ FWT) and *S. vermiculata* (2.42 nmol g^−1^ FWT) (Figure 5A). Such responses were associated with significantly higher H_2_O_2_ values in *S. schimperi* (1.16 µmol g^−1^ FWT) than *S. monoica* (0.23 µmol g^−1^ FWT) and *S. vermiculata* (0.12 µmol g^−1^ FWT) (Figure 5A). The corresponding differences in the cellular levels of antioxidants such as total phenolics, flavonoids, carotenes, betacyanin, and reduced glutathione, were also measured and compared. Our analysis revealed great differences among *Suaeda* species, with *S. vermiculata* being the lowest in flavonoids and phenolic levels (Figure 5B). Compared to *S. vermiculata*, *S. monoica* and *S. schimperi* had about 2.69- and 7.25-fold higher flavonoids and 3.0- and 8.3 -fold higher phenolics, respectively. Similarly, *S. vermiculata* had significantly lower carotenoids (0.09 mg g^−1^ FWT) than *S. monoica* (0.14 mg g^−1^ FWT) and *S. schimperi* (0.25 mg g^−1^ FWT) (Figure 5C). It also had significantly lower reduced glutathione (0.67 mmol g^−1^ FWT) than *S. schimperi* (0.98 mmol g^−1^ FWT) and *S. monoica* (1.28 mmol g^−1^ FWT). On the other hand, *S. monoica* had significantly lower betacyanin (0.69 mg g^−1^ FWT) than both *S. vermiculata* (4.42 mg g^−1^ FWT) and *S. schimperi* (17.40 mg g^−1^ FWT) (Figure 5C).

### 2.8. Correlation among the Tested Physiological Responses

**Figure 6:**
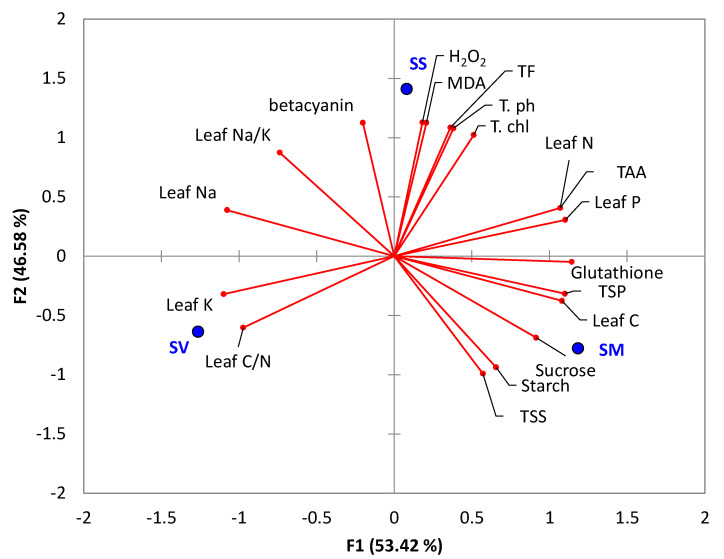
Biplot of principal component analysis of the monitored physiological attributes in the tested *Suaeda* species. SM: *S. monoica*, SV: *S. vermiculata*, and SS: *S. schimperi*. Abbreviated parameters are TSS: total soluble sugars, T. chl: total chlorophyll, T.AA: total amino acids, MDA: Malonaldehyde, TF: total flavonoids, Tph: total phenolics, and TSP: total soluble protein.

In order to assess the correlation among the various physiological attributes with the studied *Suaeda* species, we subjected a matrix of the determined parameters to principal component analysis (PCA). *Suaeda schimperi* was segregated in the upper right side of the PCA plot, where it showed a correlation to H_2_O_2_, MDA, total flavonoids, total phenolics, and total chlorophyll. However, *S. monoica* was separated on the lower right side and revealed a close correlation to sucrose, starch, TSS, leaf C, and total soluble proteins. On the other hand, *S. vermiculata* was segregated in the lower left side of the PCA plot and showed a close correlation to leaf K and leaf C/N ratio (Figure 6).

## 3. Discussion

The hyper-arid climate of the eastern coast of the Red Sea aggravates the high salinity- and PTE-induced deteriorative effects on the physiology of halophytes in the region, including *Suaeda* species. Such harmful effects are predicted to be more intense under climate change scenarios. The successful adaptation of *Suaeda* plants in such harsh environments reflects unique physiological, biochemical, and cellular adjustments that enable them to overcome salinity-induced constraints such as severe ionic, osmotic, and oxidative stresses [38]. Therefore, targeting these plants in their saline natural habitats is critical for a better understanding of their tolerance against salt stress. Herein, three *Suaeda* species that are genetically related but naturally distributed in different salt marshes in the region were selected to investigate their possible distinctive physiological adaptation against physicochemical properties of the rhizospheric soil in their natural habitats.

### 3.1. Soil Physicochemical Properties and the Relative Magnitudes of Salt Stress Imposed on the Investigated Suaeda Species

Soils supporting the investigated *Suaeda* species had relatively similar physical features (Table 1), which may not impose significant restrictions on their root growth. The tested soils also had low WHC values, which were correlated with high sand content that usually exhibits small cohesion forces to hold water molecules against gravity [39]. The relatively high WHC in soils supporting *S. vermiculata* and *S. schimperi* is attributed mainly to their high silt and clay and organic matter content, respectively (Table 1).

The rhizospheric soils of *S. vermiculata* and *S. schimperi* had significantly higher EC than that of *S. monoica* (Table 1). The high EC values in the tested arid saline habitats are attributed mainly to the synergistic interplay among the harsh metrological data in the region (high temperature, low rainfall, and increased evapotranspiration). The increased rate of evapotranspiration enhances the upward movement of water and its dissolved salts, which accumulate in the soil surface layers leading to hypersaline conditions and the observed white crust during summer [40]. The higher alkaline value of soil supporting *S. vermiculata* (8.65) is mainly correlated with the relatively higher silt and clay content of the soil, which encourages binding of metal ions (Na^+^ in particular) with a high potential of NaH_2_CO_3_. Signs of that are the higher water-soluble Na^+^ and HCO_3_^−^ concentrations in soil supporting *S. vermiculata.* The obtained EC values of the rhizospheric soil are equivalent to NaCl concentrations that are beyond the stimulatory concentrations of most *Suaeda* species [41]. This higher EC value of soil supporting *S. vermiculata* is also attributed to its higher silt and clay content, which retains higher amounts of salts onto the soil matrix. The strong variations in EC, Na^+^, and Cl^−^ among the tested rhizospheric soils indicate that the tested species experience different magnitudes of salt stress. The high soluble Mg^2+^ concentration in soil supporting *S. schimperi* is mainly due to its closeness to the Red Sea coast. The high colloidal content of soil supporting *S. vermiculata* maintained its sorption capacity of soluble ions (HCO_3_^−^, Na^+^ and K^+^).

Most soils under the tested *Suaeda* species are deficient in C, (<5%) N (<280 kg ha^−1^), and S (<10 mg kg^−1^) [42] (Table 2). Alike, phosphorus concentration showed low values according to soil fertility standards. Such soil nutrient deficiency could reduce leaf nutrient contents and thus may exacerbate the deleterious physiological effects of salinity. Along with the above differences in soil salinity, the rhizospheric soils exhibited qualitative and quantitative differences in the composition of the PTEs pool (Table 3). The soil supporting *S. schimperi* had the highest Co values, whereas soil supporting *S. monoica* showed the highest Hg content. The high levels of these elements might be derived from various anthropogenic activities developed along the east coast of the Red Sea.

The complex interplay between these PTEs and salinity interferes with the uptake and transport of essential elements (nutrients) and/or PTEs [38,43,44] and thus affects their cellular levels in the tested species (Table 3). Values of most PTEs showed higher levels (mg kg^−1^) than those justified by standard regulatory bodies (WHO, FAO, and EPA): Cd (0.01), Cr (1.3), Cu (10), Fe (425), Ni (10), Pb (2), and Zn (100) [37]. Such high values of PTEs suggest a potential use of these plants for phytoremediation purposes rather than their utilization in human/animal nutrition [24,38]. Signs of that are the high bioaccumulation indices of PTEs since the studied halophytes exhibited outstanding hyperaccumulating potentials with BCR values ranging between 3.3 and 466.1 (Figure 2). These results suggest that the investigated *Suaeda* species can be used as efficient biological tools for PTEs phytoremediation in contaminated soils. The higher bioaccumulation factor of Cr could be due to the negative charge of chromate ions, which are weakly bound onto soil colloids and are easily taken up by plants.

### 3.2. Utilization of Inorganic Ions as “Cheap” Osmotoica in the Tested Suaeda Species

Accumulation of inorganic salts as energy cost-effective osmotica is a relevant strategy of salt tolerance in many halophytes [45,46]. In the current study, the tested species accumulated high levels of cations, particularly Na^+^ and K^+^ (Table 4). The active transport of such high levels of inorganic salts to leaves of the tested species may indicate a constitutive mechanism for osmotic adjustment [46]. Consistent with that, a significant contribution of the transported ions in the aerial parts to salt tolerance in *S. fruticosa* has been reported [47]. Although dicots are known for their active transport of Na^+^ to their aerial shoot, maintaining a balanced Na^+^/K^+^ ratio in the cytoplasm is an important mechanism for salt tolerance because of the interference of Na with K uptake/accumulation and its disruptive effects on protein synthesis and activities of several cytoplasmic proteins [45,48]. In the current study, *S. monoica* had the lowest Na^+^/K^+^ ratio among species (Table 4), suggesting a pronounced role of such a low Na/K ratio in salt tolerance in this species similar to other halophytes such as *Limoium* species [46]. *Suaeda schimperi* and *S. vermiculata* accumulated higher Mg concentration than *S. monoica* suggesting a role of Mg in salt tolerance in these two species, possibly via interference with mRNA translation and consequently protein synthesis. Similar findings have been reported in other halophytes such as *Atriplex isatidea* and *Inula crithmoides* [49]. Interestingly, leaves of *S. schimperi* and *S. monoica* had significantly higher phosphorus than *S. vermiculata*. Along with being a major component of many primary and secondary cellular molecules [46], P has been implicated in improving salt tolerance via improving physiological mechanisms that promoted the full recovery of stressed plants [50]. It is worth mentioning that the tested species maintained relatively similar levels of Ca^2+^, which is known for its role in maintaining Na^+^ and K^+^ homeostasis via the Salt Overly Sensitive pathway [51].

The observed variation in elemental concentration in leaves of the tested species was associated with their known succulence, particularly in *S. vermiculata*, which had more succulent leaves than the other two species (Figure S1). The succulence phenotype in *S. vermiculata* was associated with its higher foliar Na^+^ level. These results are consistent with those of [52], who indicated that Na^+^ contributes significantly to succulence phenotype. In addition, its higher foliar ionic content can contribute to building up a gradient in the osmotic potential, which allows *S. vermiculata* to take up more water to secure efficient osmotic adjustment to overcome the low external water potential. The differences in leaf succulence among the tested species may reflect comparable differences in cell size at the tissue level as well as leaf anatomy [52]. Many succulents are described as salt accumulators and are considered the most salt-tolerant because of their succulence, which enables them to have higher vacuolar concentrations of Na^+^ and Cl^−^ than external concentrations and to avoid desiccation in dry soil [53]. Succulent species with high leaf C may improve the energy returns from carbon investment for cellular components favoring salt tolerance.

### 3.3. Relative Physiological Responses of the Tested Suaeda Species in Response to Their Soil Microenvironment

The variation in soil salinity and PTEs in the current study area are expected to negatively impact most aspects of plant growth and physiology. The tested species differed significantly in their C assimilation and its related physiological traits. Despite the superiority of *S. schimperi* in photosynthetic pigments (Chl a, Chl, b, and total Chl.; Figure 3A), *S. monoica* tended to have consistently higher averages TSS, sucrose, starch, total carbohydrates, and total leaf C than the other two species (Figure 3B, Table 4). The higher carbohydrates and C accumulation in *S. monoica* may reflect its higher efficiency in C assimilation/sequestration and can be attributed to several reasons: its higher leaf N and leaf P (Table 4), which are critical nutrients for gas exchange and C assimilation as well as other cellular fundamental activities [53]. A positive correlation between the foliar leaf N and/or P and photosynthetic efficiency as well as the greater stochiometric homeostasis of leaf N in N-deficient soils have been reported [53,54]. According to the PCA and correlation analyses, a significant positive correlation (r ≈ 0.99) was recoded between leaf N with leaf P and total amino acids (Table S3, Supplementary Data). Likewise, leaf C/N exhibited a significant positive correlation with leaf P and total amino acids (0.96 and 0.90, respectively). Furthermore, the inappreciable levels of Cd, Hg, and Pb in leaves of *S. monoica* (Table 3) can have a positive cumulative effect on the efficiency of carbohydrates accumulation. In contrast, the high Cd and Hg in *S. vermiculata* and *S. schimperi* can significantly reduce their photosynthetic activity. For example, Cd forms mercaptide with the thiol group of RUBISCO protein and thus hinders its activity and consequently suppresses their photosynthetic efficiency, which explains their reduced carbohydrates accumulation [55]. It is worth mentioning that *S. monoica* had high levels of other PTEs such as Cr, Cu, and Ni; however, its higher carbohydrate content may suggest that the toxicity of these minerals was tolerated internally in *S. monoica* either by compartmentalization or binding these PTEs in less toxic forms [56,57]. The relatively low carbohydrate values in *S. vermiculata* and *S. schimperi* can be also attributed to the high salinity of their rhizospheric soil (Table 1) as well as their relatively higher leaf Na^+^ (Table 4), which seems to be beyond their optimum salt range and their ability of compartmentalization into vacuoles [29] and thus accumulates in the cytoplasm and exerts its inhibitory biological effects [58,59,60]. Other possible reasons for the reduced carbohydrates in *S. vermiculata* are the reduced content of photosynthetic pigments (Figure 3A), low leaf-N (Table 4), reduced carbon reductive tissue area, and high foliar ABA content [61]. In fact, down regulation of carbohydrates and other biochemical and physiological processes may be a strategy that *S. vemiculata* employs to avoid the oxidative stress it encounters in its natural habitat. It is worth mentioning that, compared to both *S. monoica* and *S. schimperi*, *S. vermmiculata* had significantly higher foliar C/N ratio (Table 4), which is an indicator on improved nitrogen use efficiency (NUE) in N-deficient soil [62]. These results fit nicely with the adaptive growth hypothesis, which suggests that “plants with higher C/N ratio promote NUE under strong N-limited conditions to ensure survival priority, whereas plants with a lower C/N ratio under less N-limited environments benefit growth priority” [15]. On the other hand, the extensive consumption of carbohydrates for building up carbon skeletons of amino acids in *S schimperi* (Figure 4) and the severe oxidative stress (Figure 5A) it encounters partially explain its reduced levels of carbohydrate.

The tested species exhibited significant variation in amino acid biosynthesis, which showed a significant positive correlation with leaf P (r = 0.97) (Table S2, Supplementary Data). *Suaeda monoica* and *S. schimperi* were more efficient in amino acids biosynthesis than *S. vermiculata* (Figure 4). *Suaeda monoica* and *S. schimperi* had a relatively similar ranking of amino acids within the pool; however, such ranking was significantly disturbed in *S. vermiculata* (Figure 4B). Glutamic and aspartic acids dominated the amino acid pool across species, reflecting their “housekeeping” functions in the three species. Interestingly, *S monoica* and *S. schimperi* accumulated significantly higher foliar proline and phenylalanine contents than *S. vermiculata* suggesting a potential role of proline as an important compatible osmolyte in both *S. monoica* and *S. schimperi* but not in *S. vermiculata*. This is consistent with the recently reported low proline level in shoots of *S. vermiculata* [28]. Proline and phenylalanine are two critical amino acids under stress. The former is an important osmolyte in many halophytes, whereas the latter is the main entry point to the phenylpropanoid pathway through which flavonoid and phenolic compounds are synthesized [63]. Consistent with that, our measurements of these secondary metabolites revealed significantly higher flavonoids and phenolic in *S. schimperi* and *S. monoica* than *S. vermiculata* (Figure 5B). In addition, our analysis suggests a significant positive correlation between flavonoids and total phenolics (r = 0.97) and betacyanin (r = 0.89) (Table S3, Supplementary Data).

### 3.4. Relative Oxidative Stress and Antioxidants Synthesis in the Tested Suaeda Species

The tested species are exposed to oxidative stress of different severity. *Suaeda schimperi* suffers the highest stress as indicated by its highest levels of MDA (Figure 5A). Such responses revealed an imbalance between the production of ROS and radical quenchers in *S. schimperi* at the cellular level [64]. Such high MDA level in *S. schimperi* is attributed to the high salinity of its rhizospheric soils (Table 1), its higher foliar level of H_2_O_2_ (Figure 5A), and the simultaneous toxicity of PTEs in their leaves, particularly Hg, Cr, and Fe (Table 3). High salinity induces ROS generation via disruption of electron transport in chloroplasts and mitochondria and thus induces lipid peroxidation [65,66]. A higher level of H_2_O_2_ can directly induce oxidative stress because of its oxidation potential or indirectly via the generation of highly reactive hydroxyl radicals via Fenton’s reaction in the presence of increased levels of transient PTEs [67]. Unfortunately, cells do not have an enzymatic system to detoxify such hydroxyl radicals [67]. Generation of leaf H_2_O_2_ correlates positively with leaf contents of total flavonoids and total phenolics (r = 0.97). Further, the level of Hg in *S. schimperi* leaves exceeded the toxic threshold of Hg in plants [68], thereby interfering with mitochondrial activity and triggering ROS generation and MDA accumulation [17,38]. Mercury (Hg) can also hinder water flow in *S. schimperi* via binding to water channel proteins and induction of stomatal closure [69]. Such a high level of MDA was reflected in its relatively low TSS, sucrose, starch, and total carbohydrates but not in the level of photosynthetic pigments suggesting that the salinity-induced oxidative stress affects photosynthetic activity rather than chlorophyll synthesis in *S. schimperi.* A positive significant correlation was recorded between total chlorophyll with H_2_O_2_ (r = 0.92), total flavonoids (r = 0.97), and total phenolics (r = 0.96) (Table S3, Supplementary Data). Unlike *S. schimperi*, the relatively low levels of MDA in leaves of *S. vermiculata* and *S. monoica* are attributed, in part, to their low levels of H_2_O_2_ (Figure 5A) and to their relatively high Zn and Pb (Table 3), which might induce antioxidant enzymes (CAT, SOD GPX) [70]. In addition, the high succulence in these two species (Figure S1) may also minimize the detrimental effects of salinity and PTEs and thus reduce their-induced oxidative stress. Further, the reduced oxidative stress in *S. vermiculata* leaves may also be attributed to salinity-induced guaiacol peroxidase activity and to its overall downregulated biochemical and physiological processes (Figure 3 and Figure 4) [28].

*Suaeda vermiculata* and *S. schimperi* accumulated 6- and 25- fold higher betacyanin than *S. monoica*, respectively (Figure 5C), suggesting a possible role of this stress-related pigment in salt tolerance in these two species. This hypothesis is supported by the reported positive correlation between *Suaeda* leaf betacyanin content and both soil salinity and leaf H_2_O_2_ [71,72]. Our results are thus consistent with the physiological roles of betacyanin in the prevention of salt toxicity [72], acting as an osmotic pigment, antioxidant, and protecting halophytes against the H_2_O_2_-induced protein oxidation [72]. In fact, a trade-off between leaf chlorophyll and betacyanin for maintaining growth and survival in saline environments has been reported in *S. salsa* (Li et al., 2018; Wang et al., 2007) and *S. japonica* (Hayakawa and Agarie, 2010). In the current study, the high level of betacyanin (Figure 5C) and the reduced level of all chlorophyll fractions (Figure 3A) in *S. vermiculata* suggest that the betacyanin/chlorophyll trade-off scenario operates in this species to minimize the salinity-induced ROS (Figure 5A). The obtained results also pointed to a significant negative correlation between leaf Na^+^ and glutathione (r = −0.93) (Table S3, Supplementary Data). In *S. schimperi*, despite its high levels of betacyanin and other measured antioxidants (carotenoids, phenolics, flavonoids, and reduced glutathione) (Figure 5B,C), it suffered from the highest magnitude of oxidative stress (Figure 5A). These results do not necessarily minimize the biological significance of these compounds in salt tolerance in *S. schimperi* but may rather indicate that the levels of these compounds are not sufficient to completely neutralize the severe salinity- and PTEs-induced ROS production in *S. schimperi* because of its very high saline rhizospheric soil (Table 1). In addition, these compounds may also be involved in other salt tolerance mechanisms such as the protection of photosynthetic pigments against degradation by salt stress in this species [73], which partially explains the high photosynthetic pigments in *S. schimperi* in the current study (Figure 3A). Therefore, *S. vermiculata* and *S. schimperi* seem to invest in betacyanin synthesis as an adaptive strategy against the severe salinity stress they encounter in their harsh environment. In fact, the leaf reddening phenotypes because of betacyanin accumulation can be easily recognized in these two species. In addition, the reduced oxidative stress in *S. vermiculata,* regardless of the high EC values and PTEs content in its rhizospheric soil, suggests that this halophyte may have optimized its growth, biochemical processes, and antioxidant defense to minimize the oxidative damage it encounters in its natural environment. This hypothesis is consistent with a recent study, which indicated that *S. vermiculata* might downregulate its biochemical and physiological processes to avoid oxidative stress [28].

## 4. Materials and Methods

### 4.1. Study Site and the Selected Suaeda Species

The current study was carried out on three *Suaeda* species (*Suaeda monoica* Forssk. ex J.F.Gmel., *Suaeda vermiculata* Forssk. ex J.F.Gmel., and *Suaeda schimperi* Moq) that naturally grow in salt marsh habitat on the eastern coast of the Red Sea at Al-Qunfudah Governorate (19°7′35.1″ N, 41°4′43.9″ E), southwest of Saudi Arabia. The habitat has typical characteristics of not flooded salt marshes. The region has a typical arid dry climate with a maximum temperature of 42.6 °C and a minimum temperature of 21 °C. In addition, the tested salt marshes receive erratic and irregular precipitation in time and quantity. Some metrological data in the study area are illustrated in Table S2 (Supplementary Data). *Suaeda monoica* is a leaf succulent 1 m height bushy shrub, whereas both *S. vermiculata* and *S. schimperi* are woody shrubs with tiny oval and cylindrical succulent leaves, respectively [53]. The *Suaeda* species were identified according to [31].

### 4.2. Plants and Soil Sampling

Six homogenous medium size representative plants from each of the dominant *Suaeda* species at the flowering stage in each of the selected salt marshes were marked, guarded, and used for collection of plant material for downstream analysis. From each individual plant, three batches of leaves (100 each) were collected, washed thoroughly with deionized water, blotted dry, and divided into two groups. The first group was frozen immediately in liquid N and transferred to −80 °C. The second group was collected in plastic bags and brought on ice to the laboratory, where their fresh weight was recorded and then dried in an electric oven at 70 °C until reaching constant dry weights. The dried leaves were then ground into homogeneous powder using a stainless-steel grinder and used for elemental analyses.

Soil samples were simultaneously collected with plant materials as described by [38]. The selected plants were uprooted; their roots were separated, put into sterile plastic bags, and brought to the laboratory. Roots were gently shaken to remove loosely attaching soil particles. Firmly root-adhering soil particles (rhizospheric soil) were air-dried at room temperature, passed through 2 mm sieve, and stored in sterile polyethylene bags for soil physicochemical analyses.

### 4.3. Soil Physicochemical and Plant Elemental Analyses

#### 4.3.1. Soil PHYSICOCHEMICAL ANALYSES

Particle size distribution of soil was determined using sieve methods according to [74]. Water holding capacity of soil was determined using the gravimetric method, hydraulic conductivity by Darcy’s law, and the soil porosity from the measured values of soil particle and bulk densities calculations [75]. Soil electrical conductivity (EC) was measured using HANNA (HI9835) EC meter in 1:2.5 soil/water extract, soil pH (1:2.5 DI water suspension) by Jenway 3505 pH/mV/Temperature Meter and the total carbonate content (expressed as CaCO_3_) using the gasometric determination following 6.0 M HCl application [76]. Water-soluble cations and anions (1:2.5 DI water extract) were determined using standard methods [76]: Na^+^ using a Sherwood, flame photometer (MODEL 360), Ca^2+^, Mg^2+^, and K^+^ using ICP-OES Thermo Scientific™ iCAP™ 7000 Plus Series, CO_3_^2−^ and HCO_3_^−^ by titration with a standardized H_2_SO_4_ solution and Cl^−^ by AgNO_3_ titration. Total organic elements concentration in soil (C, N, H, and S) was determined using dry combustion method by a Thermo Scientific Flash 2000 analyzer. Available concentrations of PTEs were determined using ICP-OES after extraction by diethylene tri-amine Penta acetic acid (DTPA).

#### 4.3.2. Plant Analyses

Plant Elemental Analysis: Fine powdered dried leaves were used for determination of elements such as C, N, H, and S using CHNS analyzer (Thermo Scientific Flash 2000) following dry combustion technique. Other inorganic elements were determined using ICP-OES in the acid-digested leaf samples. Plant samples were digested using HCl/HNO_3_ mixture (3:1 *v*/*v*) in a microwave digester (Milestone MLS 1200 Mega).

Leaf Water Content and Succulence: Leaf water content (LWC), relative to fresh weight, was calculated using the equation: LWC = [100 × (leaves fresh weight-leaves dry weight)/(leaves fresh weight)] [77]. Leaf succulence was calculated using the equation [succulence = (leaves fresh weight − leaves dry weight)/leaves dry weight] as described previously [78].

Chlorophyll Pigments: Photosynthetic pigments including chlorophyll a (Chl a), chlorophyll b (Chl b), and carotenoids in 50 mg frozen leaves were extracted in 10 mL cold aqueous acetone (80%) and their concentrations were measured spectrophotometrically at 663.2, 646.8, and 470.0 nm according to [79] and were expressed as mg g^−1^ FWT.

Determination of Betacyanin: Quantities of 500 mg powdered, frozen leaves were extracted by grinding in 20 mL cold methanol at 4 °C for 30 min. The homogenates were centrifuged at 4 °C at 10,000 rpm for 10 min. The supernatants were discarded, and the pellets were re-extracted in distilled water at 4 °C. The concentrations of betacyanin were then measured spectrophotometrically at 538 nm and calculated using the molar extraction coefficient of betacyanin of 5.66 × 10^4^ [80].

Determination of Carbohydrate Fractions: Carbohydrate residues in 100 mg powdered dry leaves were extracted in aqueous ethanol (80%). The ethanolic extracts were collected and completed to specific volumes and used for spectrophotometric determination of total soluble sugars (TSS) and sucrose using anthrone reagent at 620 nm [81]. Starch in sugar-free plant residue was extracted in perchloric acid: water (6.5:1), and the liberated sugars were then estimated using anthrone method as described previously [82]. Carbohydrate fractions were calculated using standard curves of pure glucose and sucrose and expressed as mg g^−1^ DWT.

Determination of Total Soluble Proteins and Amino Acids: Total soluble proteins (TSP) were extracted by grinding 500 mg of frozen leaves in chilled acetone to remove pigments [83]. TSP in dry precipitates was then extracted in Tris-HCl buffer (0.05 mM, pH 9.0) and then determined using coomassie brilliant blue G 250 spectrophotometrically at 595 nm [84]. TSP concentrations were calculated using bovine serum albumin standard curve and expressed as mg g^−1^ DWT. Amino acid analysis was carried out using amino acid analyzer (Biochrom 30; Biochrom Ltd., Cambridge Science Park, Cambridge, England) as described by AOAC (2012).

Assessment of Oxidative Stress in Leaves: Lipid peroxidation and hydrogen peroxide (H_2_O_2_) were monitored as key indicators of oxidative damage in leaves. Lipid peroxidation was monitored as the level of malondialdehyde (MDA) using 2-thiobarbituric acid method spectrophotometrically as described previously [85] with minor modification. Frozen leaf tissues (500 mg) were extracted in 5 mL 10% trichloroacetic acid (*w*/*v*), and the homogenates were centrifuged at 4 °C for 10 min at 4000 rpm. A total of 0.5 mL of the supernatants were mixed with 0.5 mL of thiobarbituric acid (0.6%; *w*/*v*), and the mixtures were incubated at 95 °C for 15 min, cooled on ice, and centrifuged at 4 °C for 10 min at 4000 rpm. The absorbance of the pink color was measured at 450, 532, and 600 nm. The MDA concentration was calculated using the extinction coefficient of 155 and expressed as nmol g^−1^ FWT. For H_2_O_2_, 500 mg of powdered, frozen leaf tissues were homogenized in 5 mL cold phosphate buffer (50 mM potassium phosphate, 1 mM EDTA, pH 7.5) and centrifuged at 4 °C for 15 min at 4000 rpm. The supernatants were then collected and used for the measurement of H_2_O_2_ spectrophotometrically using a hydrogen peroxide assay kit (Biodiagonistic, HP 25, Giza, Egypt) according to the manufacturer’s instructions.

Estimation of Antioxidant Substances: Powdered dry leaves (100 mg) were extracted in acetone to remove chlorophyll, dried, and resuspended in distilled water. Total flavonoids were measured spectrophotometrically using AlCl_3_ reagent and quercetin as a standard at 410 nm [86]. Total phenolics in the same aqueous extracts were determined spectrophotometrically using the Folin–Ciocalteu method and gallic acid as a standard at 760 nm [87]. The concentrations of both total flavonoids and phenolics were expressed as mg g^−1^ DWT. Reduced glutathione was extracted by homogenizing 500 mg of frozen leaf tissues in 5 mL cold potassium phosphate buffer (50 mM potassium phosphate, pH 7.5, 1 mM EDTA). The homogenates were centrifuged for 15 min at 4000 rpm and 4 °C. Supernatants were then collected and used for spectrophotometric determination of reduced glutathione using Biodiagonistic kit (GR 2511) following the manufacturer’s instructions.

### 4.4. Quality Control

Plant and soil measurements were carried out by an ISO/IEC 17025 accredited laboratory to ensure data accuracy and verification. Analytical measurements were conducted under constant temperature (25 ± 0.5 °C) with standardized protocols of replication controls and blanks. Deionized water (18.2 MΩ) (Nanopure water, Barnstead) was used for chemical solutions preparation, and all analytical grade chemical reagents (Merck-Darmstadt, Germany) were used without further purification procedures. A certified soil reference material (BIPEA, France) was used for optimization of soil analysis accuracy and verification. In addition, the accuracy of inorganic element determination was verified using Thermo Fisher Scientific standard solutions (R^2^ ≥ 0.99). Organic elements measurement was optimized by a BBOT standard (C_26_ H_26_ N_2_ SO_2_): carbon (72.52%), hydrogen (6.09%), nitrogen (6.51%), and sulfur (7.44%). The recovery values of organic and inorganic elements oscillated in the range 93.6–103.4, and the data precision was justified at maximum relative standard deviation (RSD) value ≤ 5%. Values of limit of detection (LOD) for inorganic elements (µg L^−1^) were Al (52.6), Cd (53.4), Cr (31.8), Co (37.2), Cu (20.8), Fe (46.8), Hg (23.5), Mn (29.7), Ni (40.2), Pb (54.9) and Zn (40.9).

### 4.5. Statistical Analysis

ANOVA analysis for the studied parameters was performed using COHORT/COSTAT software (798 Lighthouse Ave. PMB 329, Monterey, CA, USA) using the least significant difference test (LSD) at the significance level of 95%. In addition, data (Figure 2) were also presented in box and whisker plots using OriginPro 9.1: mean (dot), median (center line), lower quartile (lower border of the box), and upper quartile (upper border of the box). Pearson’s correlations coefficients were applied to study the relationships among different physiological responses. Principal component analysis (PCA) was carried out using XLSTAT statistical computer software package, version 14 (Addinsoft, New York, NY, USA).

## 5. Conclusions

The unique genetic and physiological characteristics of halophytes support their high potential utilization as promising biological resources for improving the world’s agriculture under climate change scenarios. This investigation deliberates on the premise that studying biochemical and physiological features of *Suaeda* species in their natural environments will support our planning for the future management of agricultural practices, especially in arid climate conditions. The key findings of the current investigation can be summarized as:

- *Suaeda* species are exposed to varying levels of salinity stress along with nutrient stress either as deficiency of essential nutrients such as N, K, P, or as elevated levels of PTEs.
- *Suaeda* species employ different and efficient adaptive strategies to maintain cellular homeostasis against increased levels of salinity in their rhizospheric soils.
- The high accumulation potential of PTEs, based on the bioaccumulation index of the tested *Suaeda* species, highlights their potentiality as efficient phytoextractors of soil pollutants.
- The obtained differences among the tested *Suaeda* species in the current study are driven mainly by species-specific tolerance strategies, and such specificity is shaped by the level of salinity and the genetic constitution of halophytic species.
- In essence, the obtained results of this investigation fulfill the proposed specific objectives and support the set hypotheses of the current study.

## Figures and Tables

**Table 1:** Physicochemical analyses of the investigated soils. Shown are the means of three biological replicates ± standard deviation.

Physicochemical Parameters			*S. monoica*	*S. vermiculata*	*S. schimperi*
Soil physical properties	Particle size distribution (%)	Sand	92.6	76.0	95.8
		Silt and clay	6.9	23.3	3.7
	Texture		Sandy	Sandy clay loam	Sandy
	Water holding capacity (%)		31.98 ± 3.05	38.15 ± 3.52	35.02 ± 3.21
	Porosity (%)		39.98 ± 3.53	49.93 ± 4.57	43.89 ± 3.93
Soil chemical properties	EC (dS m^−1^)		5.04 ± 0.55	18.37 ± 1.91	16.25 ± 1.04
	pH		8.10 ± 0.44	8.65 ± 0.38	7.78 ± 0.24
	CaCO_3_ (%)		0.55 ± 060	0.79 ± 0.66	0.79 ± 0.30
	Water soluble anions (Cmol/100 g)	HCO_3_^−^	5.57 ± 0.60	6.35 ± 0.66	5.33 ± 0.30
		Cl^−^	6.46 ± 0.67	13.76 ± 3.28	14.29 ± 2.29
	Water soluble cations (Cmol/100 g)	Na^+^	2.51 ± 0.15	5.49 ± 0.23	4.89 ± 0.30
		K^+^	0.46 ± 0.09	1.10 ± 0.12	0.98 ± 0.10
		Ca^2+^	7.20 ± 0.39	6.15 ± 0.41	7.60 ± 0.44
		Mg^2+^	1.79 ± 0.08	5.66 ± 0.69	10.42 ± 0.89

**Table 2:** Total organic elements, available inorganic nutrients, and available sodium in the soil surface layer of the studied locations. Shown are the means of three biological replicates ± standard deviation. ND indicates that the element was under its detection limit.

Elemental Concentrations		*S. monoica*	*S. vermiculata*	*S. schimperi*
Available nutrients (mg kg^−1^)	P	8.17 ^a^ ± 0.38	9.40 ^a^ ± 0.44	6.53 ^b^ ± 31
	K	178.8 ^b^ ± 6.2	427.8 ^a^ ± 15.4	383.8 ^a^ ± 19.0
	Ca	1441.0 ^a^ ± 127.0	1229.9 ^b^ ± 114.9	1519.1 ^a^ ± 145.9
	Mg	215.3 ^c^ ± 6.3	679.4 ^b^ ± 3.7	1250.2 ^a^ ± 13.3
Available Na^+^ (mg kg^−1^)		578.3 ^c^ ± 45	1262.1 ^a^ ± 124.3	1103.5 ^b^ ± 108.2
Total organic elements (%)	C	N.D.	0.23 ^b^ ± 0.07	1.95 ^a^ ± 0.19
	N	N.D.	N.D.	0.18 ± 0.13
	H	0.23 ^b^ ± 0.03	0.51 ^a^ ± 0.04	0.593 ^a^ ± 0.04
	S	N.D.	N.D.	0.47 ± 0.03

Means followed by the same letter are not significantly different at the probability level of 5% according to LSD.

**Table 3:** PTEs concentration in the soil surface layer of the studied locations and in leaves of the tested *Suaeda* species. Shown are the means of three biological replicates ± standard deviation. ND indicates that the element was under its detection limit.

Toxic Elements	*S. monoica*		*S. vermiculata*		*S. schimperi*	
	Concentration (mg kg^−1^)					
	Soil	Plant	Soil	Plant	Soil	Plant
Cd	ND	ND	0.024 ^b^ ± 0.002	0.496 ^a^ ± 0.055	0.112 ^a^ ± 0.011	0.499 ^a^ ± 0.058
Cr	0.380 ^a^ ± 0.038	177.108 ^a^ ± 15.591	0.286 ^b^ ± 0.029	18.668 ^c^ ± 2.272	ND	50.166 ^b^ ± 5.262
Co	0.279 ^b^ ± 0.028	6.734 ^a^ ± 0.662	0.121 ^c^ ± 0.012	5.032 ^b^ ± 0.526	0.577 ^a^ ± 0.057	3.516 ^c^ ± 0.377
Cu	2.070 ^b^ ± 0.391	75.125 ^a^ ± 7.873	2.160 ^b^ ± 0.743	17.040 ^b^ ± 1.635	2.566 ^a^ ± 0.056	19.488 ^b^ ± 2.002
Fe	5.019 ^c^ ± 0.441	902.942 ^c^ ± 85.291	8.147 ^b^ ± 0.762	1286.0 ^b^ ± 113.4	10.643 ^a^ ± 1.082	2038.4 ^a^ ± 188.6
Hg	1.813 ^a^ ± 0.155	ND	1.367 ^b^ ± 0.123	29.229 ^a^ ± 2.545	1.456 ^b^ ± 0.147	16.871 ^b^ ± 1.132
Mn	11.884 ^b^ ± 0.295	146.304 ^b^ ± 15.319	13.511 ^b^ ± 0.576	102.29 ^c^ ± 11.52	29.237 ^a^ ± 1.2211	166.053 ^a^ ± 14.973
Ni	1.623 ^a^ ± 0.056	16.852 ^a^ ± 1.981	0.441 ^b^ ± 0.020	4.518 ^b^ ± 0.503	0.066 ^c^ ± 0.013	5.964 ^b^ ± 0.654
Pb	3.812 ^b^ ± 0.147	ND	5.617 ^a^ ± 0.216	18.651 ^a^ ± 2.342	ND	9.918 ^b^ ± 1.119
Zn	1.992 ^c^ ± 0.069	113.735 ^a^ ± 11.109	5.633 ^a^ ± 0.071	74.080 ^c^ ± 8.148	3.233 ^b^ ± 0.063	93.904 ^b^ ± 10.077

Means followed by the same letter are not significantly different at the probability level of 5% according to LSD.

**Table 4:** Elemental concentration in leaves of the tested *Suaeda* species (mg g^−1^ DWT). Shown are the mean values of three biological replicates. ND indicates that the element was under its detection limit.

Elements Concentration (mg g^−1^ DWT)	*S. monoica*	*S. vermiculata*	*S. schimperi*
P	1.485 ^a^ ± 0.156	0.452 ^c^ ± 0.126	1.275 ^b^ ± 0.130
Ca	10.38 ^b^ ± 1.15	10.82 ^ab^ ± 1.22	12.63 ^a^ ± 1.27
Mg	4.99 ^b^ ± 0.51	6.51 ^a^ ± 0.71	6.74 ^a^ ± 0.63
K	10.04 ^b^ ± 0.87	12.69 ^a^ ± 1.33	10.55 ^b^ ± 1.19
Na	11.01 ^b^ ± 1.15	17.84 ^a^ ± 1.85	16.19 ^a^ ± 1.75
Na/K ratio	1.097 ± 0.045	1.407 ± 0.002	1.536 ± 0.009
C	322.56 ^a^ ± 33.22	245.76 ^b^ ± 24.58	265.16 ^b^ ± 24.25
N	32.04 ^a^ ± 3.65	11.96 ^c^ ± 1.60	29.79 ^b^ ± 4.03
S	0.377 ^a^ ± 0.031	0.357 ^a^ ± 0.025	N.D.
C/N ratio	10.07	20.55	9.09

Means followed by the same letter are not significantly different at the probability level of 5% according to LSD.

## Data Availability

Not applicable.

## Supplementary Materials

The following are available online at https://www.mdpi.com/article/10.3390/plants11040537/s1, Table S1: Ecological Risk Assessment Parameters and Their Contamination Levels. Table S2: Mean values of Metrological data in the study area. Table S3: Correlation values among physiological attributes in the tested *Suaeda species.*
