# Supplementary material for: Physiological Adaptation of Three Wild Halophytic Suaeda Species: Salt Tolerance Strategies and Metal Accumulation Capacity
Source: Plants (Basel). 2022 Feb 17;11(4):537. doi: 10.3390/plants11040537 (PMC8877964; doi:10.3390/plants11040537)
Supplement: Supplementary file 1 [file plants-11-00537-s001.zip › plants-1564527-supplementary.pdf]

Supplementary Table (S1): Ecological Risk Assessment Parameters and Their Contamination Levels.

Ecological risk assessment parameters	Value	Contamination levels
**Geo-accumulation index (I_{geo})**	$0 < I_{geo} < 1$	Uncontaminated
	$1 < I_{geo} < 2$	Slightly contaminated
	$2 < I_{geo} < 3$	Moderately contaminated
	$3 < I_{geo} < 4$	Moderately to highly contaminated
	$4 < I_{geo} < 5$	Highly contaminated
	$5 > I_{geo}$	Extremely contaminated
**Enrichment factor (E_f)**	$0 < E_f < 1$	No enrichment
	$1 < E_f < 3$	Minor enrichment
	$3 < E_f < 5$	Moderate enrichment
	$5 < E_f < 10$	Moderate to severe enrichment
	$10 < E_f < 25$	Severe enrichment
	$25 < E_f < 50$	Very severe enrichment
**The contamination factor (Cf)**	$C_F < 2$	Minimal contamination
	$2 \leq C_F < 5$	Moderate contamination.
	$5 \leq C_F < 20$	Significant contamination
	$20 \leq C_F < 40$	Very high contamination
	$C_F > 40$	Extremely high contamination
**Pollution Load Index (P_{LI})**	$0 < P_{LI} \leq 1$	Unpolluted
	$1 < P_{LI} \leq 2$	Moderately to unpolluted
	$2 < P_{LI} \leq 3$	Moderately polluted
	$3 < P_{LI} \leq 4$	Moderately to highly polluted
	$4 < P_{LI} \leq 5$	Highly polluted
	$P_{LI} > 5$	Very highly polluted
**Contamination degree (C_D)**	$C_D < 7$	Low degree of contamination
	$7 \leq C_D < 14$	Moderate degree of contamination
	$14 \leq C_D < 28$	Considerable degree of contamination
	$C_D \geq 28$	Very high degree of contamination
**Modified degree of contamination (mCD)**	mCD < 1.5	Very low contamination
	1.5 ≤ mCD < 2.2	Low contamination
	2.2 ≤ mCD< 4.4	Moderate contamination
	4.4 ≤ mCD < 8.8	High contamination
	8.8 ≤ mCD < 16.16	Very high contamination
	16.16 ≤ mCD < 32	Extremely high contamination
	mCD ≥ 32	Ultra high contamination
**Potential ecological risk index (E_r^i)**	$E_r^i < 40$	low risk
	$40 \leq E_r^i < 80$	Moderate risk
	$80 \leq E_r^i < 160$	Considerable risk
	$160 \leq E_r^i < 320$	High risk
	$320 \leq E_r^i$	Very high risk

Supplementary Table (S2): Mean values of Metrological data in the study area

Months	Min. Temperature (°C)	Max. Temperature (°C)	Avg. Temperature (°C)	Precipitation (mm)
**January**	21	31.3	26.1	24
**February**	21.4	32.2	26.8	18
**March**	23.2	34.7	28.9	15
**April**	25	37.8	31.4	15
**May**	27.2	40.7	33.9	16
**June**	29	42.6	35.8	7
**July**	30.1	41.8	35.9	8
**August**	29.9	41	35.4	18
**September**	27.9	40.9	34.4	20
**October**	25.2	38.4	31.8	12
**November**	23.6	35.3	29.4	9
**December**	21.6	32.8	27.2	29

Supplementary Table (S3): Correlation values among physiological attributes in the tested *Suaeda species*.

	TSS	Sucrose	Starch	T. Chl.	Leaf N	Leaf C	Leaf C/N	Leaf Na	Leaf K	Leaf K/Na	Leaf P	Protein	T.AA	MDA	H_2O_2	TF	TPh	Glutathione
**Sucrose**	0.54																	
**Starch**	0.35	0.49																
**T. Chl.**	-0.33	-0.14	-0.41															
**Leaf N**	0.20	0.50	0.01	0.72														
**Leaf C**	0.35	0.61	0.83	0.04	0.45													
**Leaf C/N**	0.09	-0.40	0.17	-0.78	-0.93	-0.23												
**Leaf Na**	-0.49	-0.74	-0.75	-0.07	-0.56	-0.97	0.33											
**Leaf K**	-0.14	-0.35	-0.46	-0.47	-0.59	-0.85	0.40	0.83										
**Leaf K/Na**	-0.67	-0.86	-0.78	0.40	-0.30	-0.73	0.12	0.78	0.31									
**Leaf P**	0.21	0.59	0.09	0.67	0.99	0.52	-0.96	-0.63	-0.61	-0.39								
**Protein**	0.57	0.87	0.41	0.20	0.80	0.66	-0.67	-0.79	-0.50	-0.79	0.86							
**T.AA**	0.17	0.47	0.08	0.72	0.98	0.53	-0.90	-0.61	-0.67	-0.31	0.97	0.79						
**MDA**	-0.35	-0.38	-0.57	0.69	0.44	-0.24	-0.50	0.26	-0.08	0.51	0.37	-0.05	0.45					
**H2O2**	-0.54	-0.42	-0.55	0.92	0.48	-0.15	-0.58	0.18	-0.32	0.65	0.41	-0.11	0.49	0.82				
**TF**	-0.47	-0.32	-0.42	0.97	0.61	-0.02	-0.69	0.04	-0.40	0.51	0.54	0.03	0.63	0.76	0.97			
**TPh**	-0.49	-0.27	-0.39	0.96	0.59	0.04	-0.67	-0.02	-0.49	0.50	0.53	0.04	0.61	0.73	0.97	0.97		
**Glutathione**	0.30	0.68	0.58	0.37	0.77	0.89	-0.61	-0.93	-0.85	-0.64	0.81	0.82	0.82	-0.02	0.10	0.27	0.29	
**betacyanin**	-0.62	-0.68	-0.64	0.80	0.21	-0.39	-0.34	0.44	-0.09	0.84	0.11	-0.40	0.21	0.71	0.92	0.89	0.86	-0.18

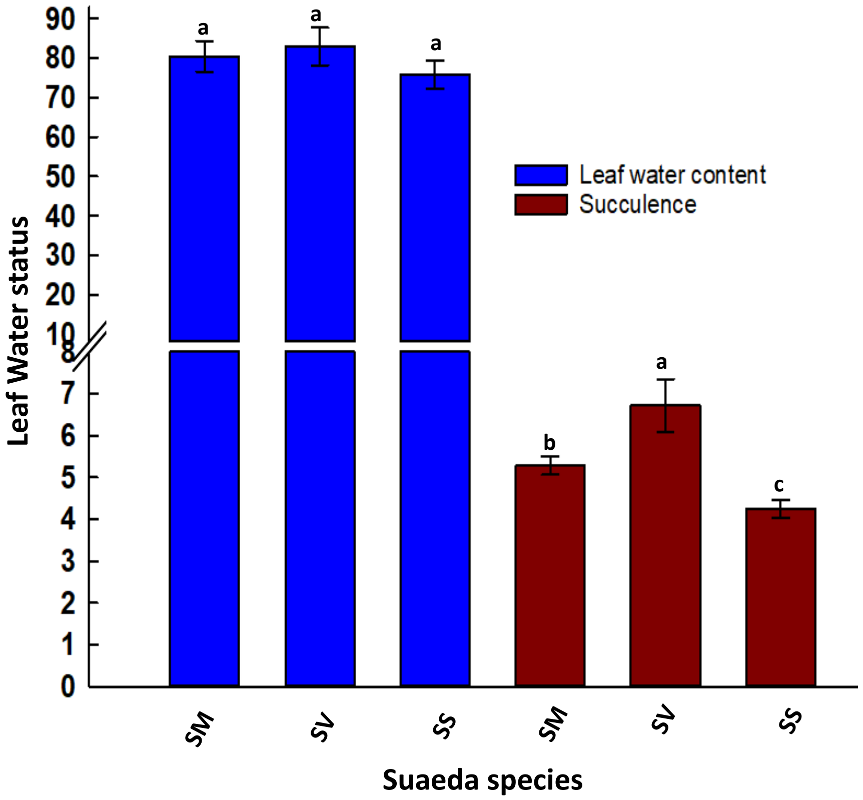

Supplementary Figure S1. Leaf water content and leaf succulence. Shown are the mean values of three .biological replicates. SM: *S. monoica*, SV: *S. vermiculata*, and SS: *S. schimperi*
